# Intensity instability and correlation in amplified multimode wave mixing

**DOI:** 10.1038/s41598-022-19051-5

**Published:** 2022-08-30

**Authors:** Haechan An, Hal Owens, Hamza Ather, Ali Shakouri, Mahdi Hosseini

**Affiliations:** 1 Birck Nanotechnology Center and Purdue Quantum Science and Engineering Institute, Elmore Family School of Electrical and Computer Engineering, Purdue University, West Lafayette, IN 47907 USA; 2 Department of Physics and Astronomy, Purdue University, West Lafayette, IN 47907 USA

**Keywords:** Atom optics, Nonlinear optics, Quantum optics

## Abstract

The dynamics of optical nonlinearity in the presence of gain and feedback can be complex leading to chaos in certain regimes. Temporal, spectral, spatial, or polarization instability of optical fields can emerge from chaotic response of an optical $\chi ^{(2)}$ or $\chi ^{(3)}$ nonlinear medium placed between two cavity mirrors or before a single feedback mirror. The complex mode dynamics, high-order correlations, and transition to instability in these systems are not well known. We consider a $\chi ^{(3)}$ medium with amplified four-wave mixing process and study noise and correlation between multiple optical modes. Although individual modes show intensity instability, we observe relative intensity noise reduction close to the standard quantum noise, limited by the camera speed. We observe a relative noise reduction of more than 20 dB and fourth-order intensity correlation between four spatial modes. More than 100 distinct correlated quadruple modes can be generated using this process.

## Introduction

Nonlinear optical media are being investigated for the generation of quantum optical states in both continuous variable and discrete variable regimes. The $\chi ^{(2)}$ nonlinearity in certain crystals can be used to generate squeezed states of light^1^ and two-photon correlations^2^ via the parametric down-conversion process. The $\chi ^{(3)}$ nonlinearity can also be used to generate bright or vacuum squeezed states of light in multiple spatial modes^3–7^.

It is known that nonlinear optical media inside resonators can exhibit instabilities in space and time as a manifestation of chaotic response of nonlinearity in presence of feedback. Above some gain threshold, the feedback provided by the resonator can lead to a chaotic response or bi-stabilities in some degrees of freedom^8–10^. The $\chi ^{(3)}$ nonlinearity that emerges from the multi-wave mixing process was previously considered in this context^11^, where temporal and spatial instabilities have been observed for transverse modes. An effective resonator can be created from counter-propagating pumps. In this way, the Bragg grating induced by the standing-wave pump^12–15^, supports amplified multi-wave mixing (AMWM) for distinct spatial modes. Above certain gain values, temporal and spatial instabilities of the individual Stokes and anti-Stokes modes can be observed^10^. Although the dynamic and dependency on experimental parameters are complex, the mathematical derivation of instability against transverse perturbations is straightforward^16^. Nevertheless, the nature of the dynamic and static instabilities, and multimode correlations that appear, despite the intensity instability of individual modes, is not well understood. The sensitivity of the process to the initial conditions, size, power etc, can enable investigation of spatial and spectral phase transitions^17–19^ for sensing and optical switching applications in both classical and, in principle, quantum regimes.

**Figure 1:**
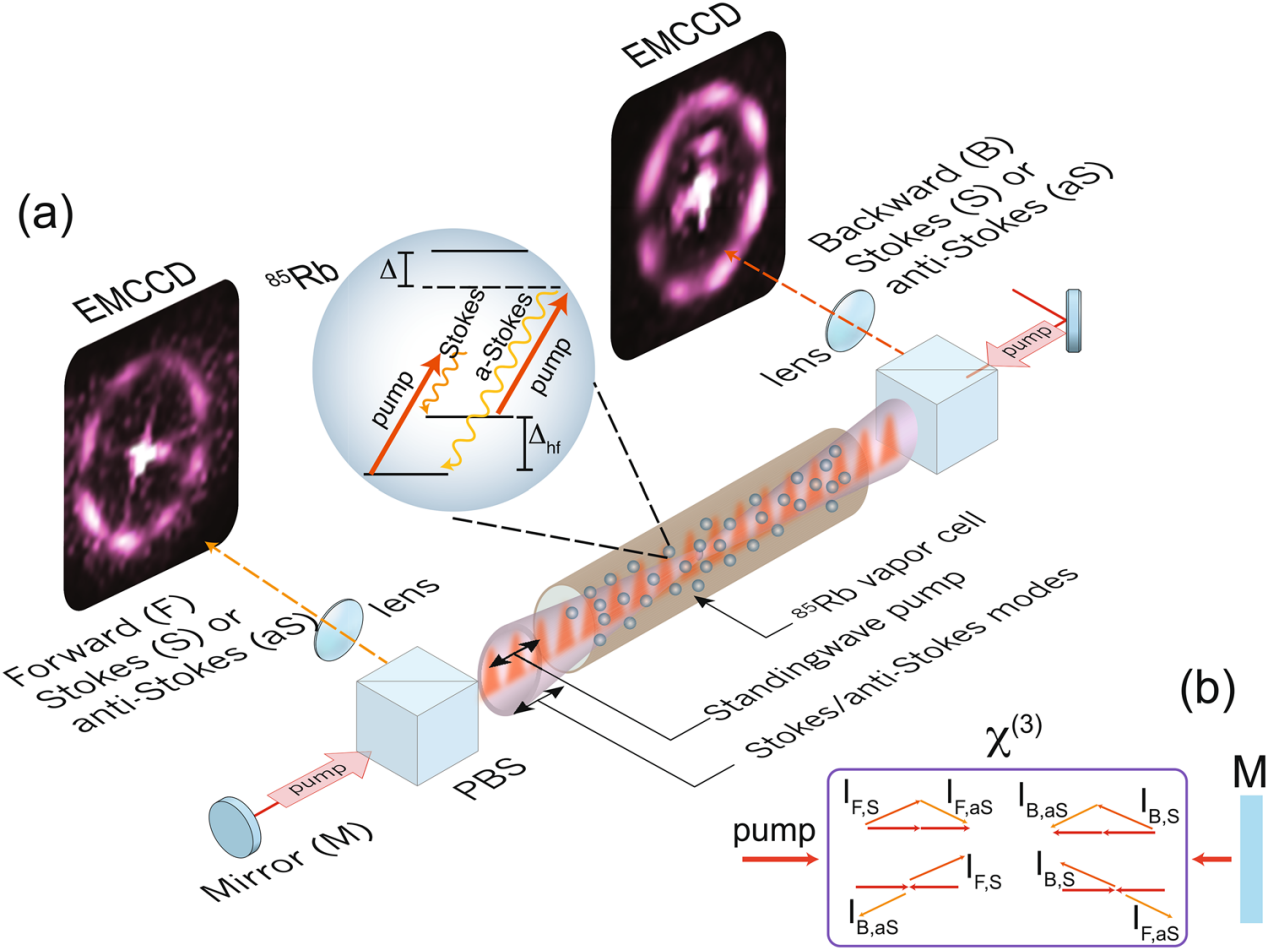
(**a**) Schematic of the experimental setup and images of correlated modes. A retro-reflected pump creates a standing wave and thus a Bragg grating inside a $^{85}$Rb vapor cell. Rb atoms interact with counter-propagating pumps and generate forward- and backward-propagating Stokes and anti-Stokes light, as shown by the energy-level structure of $^{85}$Rb. Polarization beam splitters (PBS) are used to separate linearly polarized pump from the ring-shaped scattered light. The coherently scattered light is imaged in the Fourier plane on an EMCCD camera. The images of the forward- and backward-propagating scattered modes are shown as recorded with exposure times of 10 $\upmu$s and 30 $\upmu$s. The bright spots in the middle of the images are due to the pump leakage. (**b**) A $\chi ^{(3)}$ medium with a single feedback mirror can support amplified four-wave mixing for both forward and backward-propagating Stokes and anti-Stokes modes, for which phase matching diagram is shown using subscripts (*F*, *S*), (*B*, *S*), (*F*, *aS*), and (*B*, *aS*) respectively.

A counter-propagating pump creates an atomic Bragg reflector while the multi-wave mixing process is primarily amplified for atoms with zero velocity. As the result, Stokes and anti-Stokes photons are generated symmetrically with respect to the pump axis (see Fig. 1). We observe that in some detuning regimes, a flower-like scattering pattern^20^ of Stokes and anti-Stokes light emerges with signatures of instability. The instability can be static (constant spatial patterns in time) or dynamic (oscillating patterns in time). Although the scattering patterns are instable, the Stokes and anti-Stokes modes can still be correlated. Two-mode correlation of this kind has been shown using AMWM process in an Na vapor cell^11^. Here, we study amplified four-wave mixing between multiple spatial modes (or AMWM) in Rb vapor and characterize the correlation using single-photon Electron-multiplying CCD (EMCCD) cameras. The camera enables us to simultaneously study correlation, noise and instability in the counter-propagating modes.

A schematic of the experimental setup is illustrated in Fig. 1. A 10 cm atomic vapor cell containing $^{85}$Rb atoms was heated to about 95 $^{\circ }$C and enclosed by a magnetic $\upmu$-metal shielding. A pump laser beam of wavelength about 795 nm was retro-reflected to serve as the counter-propagating pump inside the cell. The pump was typically red-detuned from the transition frequency between the lower ground state (5$^{2}$S$_{1/2}$, F=2) and the excited state (5$^{2}$P$_{1/2}$). The pump detuning from the resonance, $\Delta$, was typically in the range of 0.6 GHz $\le \Delta /2\pi \le 1.5$ GHz, generating Stokes and anti-Stokes scattering. The pump beam was collimated to 1/e$^{2}$ beam diameter of 3.6 mm with a power in the range of 200–325 $\upmu$W. The linearly polarized pump was pulsed with an acousto-optic modulator (AOM). The reason for pulsing the pump light as opposed to using continuous pump was to avoid artifact in images caused by the camera. Excessive illumination of light during frame-transfer process, which is the time between the exposure time of two adjacent frames, induces false bright vertical streaking line on the detected image. Two polarization beam splitters (PBS) were used to filter the Raman scattered light from the pump. Additional filtering was achieved by Glan-Thompson polarizers and dark-spot spatial filters.

The ring-shaped counter-propagating scattered light was collected from the two ends of the atomic vapor cell and then detected using two areas of one EMCCD camera. To have the detection area independent of the propagation length, an image of the scattered light was created in the Fourier plane. The EMCCD camera had single-photon sensitivity with an active area of 128 × 128 pixels, dark noise 0.02 *e*/*s* and exposure time as low as 10 $\upmu$s. Typically in our experiment, about 1000 images of the scattered modes were recorded under the exposure time of 10 $\upmu$s and an average frame rate of 4 ms.

For long camera exposure times, the scattered ring intensity appeared continuous in space, but for short exposure times a periodic pattern in the scattered intensity was observed. We associated the fringes in the scattered intensity captured at low exposure times to the spatial instability of AMWM^9,10,20^.

We observed that the ring-shaped scattering appeared around three regions of pump detunings divided by the two optical transitions of Rb. The scattering signal could not be observed outside these regions. At certain pump detunings, the two Raman transitions (between pump and Stokes photons and pump and anti-Stokes photons) interfere and the amplification of the anti-Stokes photons occurs. We observed that as the pump frequency was tuned away from these resonances, the scattering intensity decreased until the signal was no longer visible. In what follows, we set the pump frequency to be red-detuned ($\Delta \simeq 2\pi \times 1$ GHz) from $F=2\rightarrow F'=2$ transition. The scattering was observed for a range of $\pm 0.5$ GHz around this detuning.

**Figure 2:**
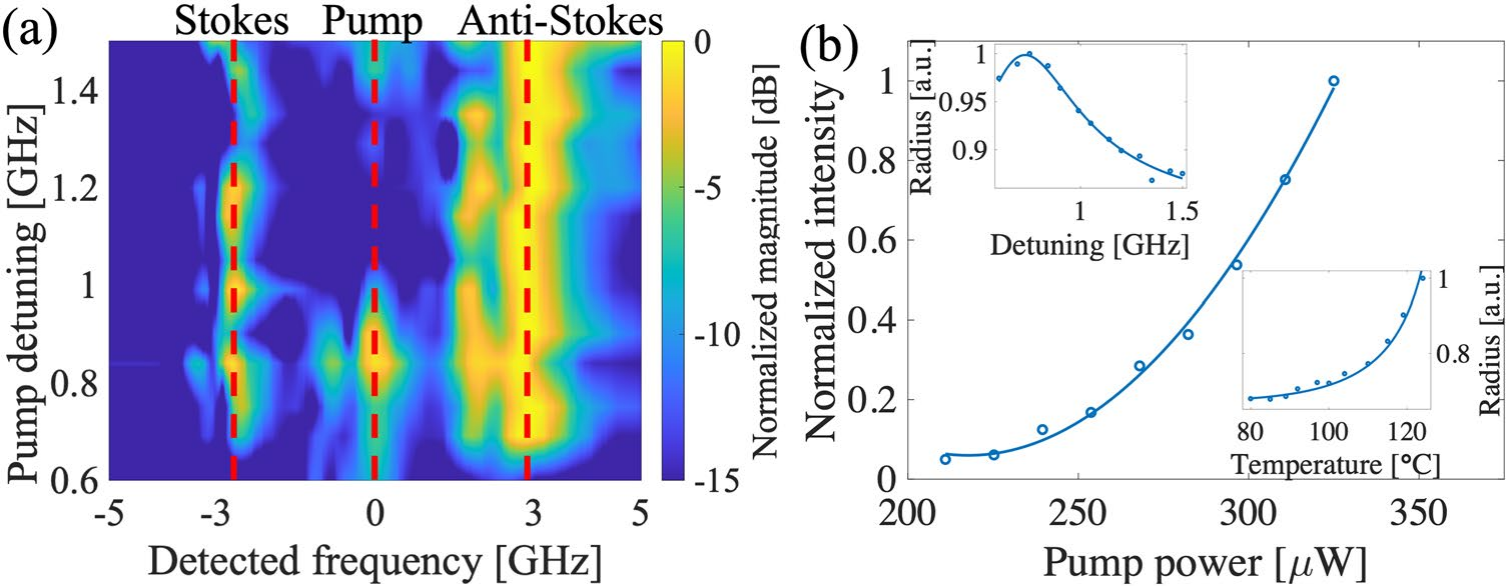
(**a**) Frequency spectrum of detected scattering signal for different pump detunings. The frequency is measured relative to the pump frequency. Red dashed lines indicate center frequencies of Stokes, pump, and anti-Stokes signals. (**b**) Scattering intensity within the ring region shows quadratic dependency with the pump power. Insets show the radius of the ring as a function of the pump detuning and the atomic cell temperature. The solid lines in the insets are theoretical fits using $R^2\approx A+B\delta t+C\delta t^2$. The models used for detuning- and temperature-dependency of delay are $\delta t=\Delta /(\Delta ^2+b)$ and $\delta t=10^{-T_0/T}$, respectively, where $T_0=4040$ K for $^{85}$Rb atoms^21^. *A*,  *B*,  *C*,  and *b* are fitting parameters.

The coherent Raman scattering takes place when the energy and phase matching conditions are satisfied for the pump, Stokes and anti-Stokes photons. The energy conservation indicates that the frequency difference between the pump light and the scattered light for zero-velocity-class atoms should be equal to the hyperfine splitting of the Rb ground-state energy levels, i.e. $\Delta _{hf}\sim \pm 2\pi \times 3$ GHz. This is confirmed by measuring the relative frequencies between the pump and different regions of the rings using an etalon filter (see Fig. 2a). It can be seen that the relative frequency of the pump and the scattered light is fixed regardless of the pump detuning.

In our case, the amplified scattering gives rise to the emergence of bright ring-shaped images of counter-propagating light. We experimentally observed that the intensity of the scattering ring changes quadratically with the pump power (Fig. 2b). We also note, that no ring-shaped scattering was observed with a single propagating-wave pump. To satisfy the phase matching condition, the angle of the scattered light in each direction relative to the pump’s propagation direction is determined as $\phi ^2\approx A+B\delta t+C\delta t^2$, where *A*,  *B*,  *C* are constants and $\delta t$ is the relative delay between the Stokes and anti-Stokes photons caused by the different refractive indices experienced by the two photons. The radius of the ring shows the transverse component of the scattered light’s momentum, which is directly related to the propagation angle of the scattered signal, $\phi$. The phase matching condition can be satisfied for a pair of co-propagating or counter-propagating photons and for a range of modes with circular symmetry (different angles, $\theta$, in the transverse plane). As $\delta t$ varies with the optical density and detuning, one can model the ring radius in terms of these experimental parameters. The model agrees well with the experimental data shown in Fig. 2b. Different pump frequencies lead to different detunings of Stokes and anti-Stokes signals from the corresponding atomic transitions. As the result, the Stokes and anti-Stokes photons experience different refractive indices. Decreasing the pump detuning and increasing the temperature leads to higher effective optical density and also larger delay, $\delta t$.

We also observed that replacing the 10 cm atomic vapor cell with a 1.2 cm cell causes the ring radius to increase by a factor of two. This can be explained from the relationship between the ring radius and distance, *d*, between the cell and the feedback mirror^16,20^. The interference between the counter-propagating beams determines the size and shape of the scattered light. At the phase matching condition, the transverse wave vector of the scattered light is inversely proportional to the square root of the distance *d*. Using a longer cell increases the optical path length and thus the effective distance between the cell and the feedback mirror. Taking the ring radius to be approximately inversely proportional to $\sqrt{d+(n-1)L}$, where *d* is around 20 cm, *n* is the refractive index of the cell, and *L* is the length of the cell, we expect the radius to reduce by a factor of less than 3 when *L* changes from 1.2 to 10 cm. This is in agreement with the observed radius change of factor 2.

**Figure 3:**
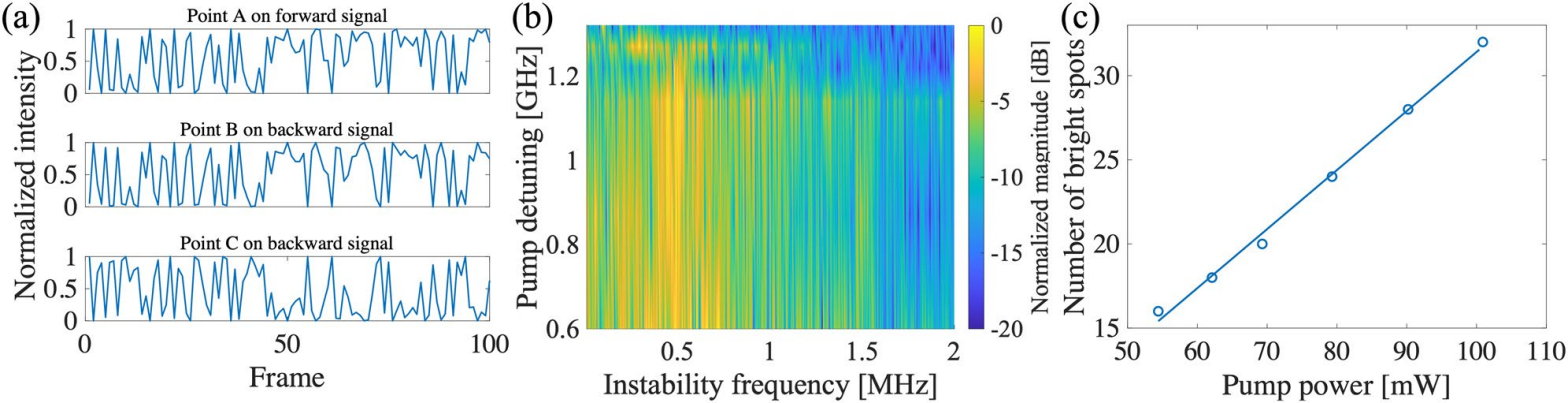
(**a**) Raw frame-by-frame intensity fluctuation of three camera pixels (A, B, and C pixels) in forward and backward directions. Pixels A and B show intensity correlation while pixel C shows anti-correlation. (**b**) Frequency spectrum of a single camera pixel for different pump detuning shows dynamic instability of scattering intensity. (**c**) Spatial frequency of the scattering ring obtained by counting the number of bright spots around the ring plotted as a function of pump power that shows linear dependency, in agreement with the theory (see text).

The ring-shaped scattering signal, created above the threshold excitation power, exhibits instabilities in space and time. The spatial instability is seen as bright and dark fringes around the ring and temporal instability is seen as oscillation between the dark and bright points in time. Figure 3a shows the raw data for the ring intensity on three pixels of the camera. Points A and B are opposite pixels on forward- and backward-propagating rings that shows intensity correlation. Point C on the backward-propagating signal shows intensity anti-correlation with the other two pixels. We note that the quantum theory of such bi-stability was previously derived for the case of a coherently driven dispersive cavity^22^. Figure 3b shows the temporal instability where the frequency spectrum of the measured AMWM signal is plotted for different pump detunings. The scattered signal with intensity instability showing broad continuous power distribution and multiple oscillation frequencies suggests a quasi-periodic transition mechanism to instability^10^.

The spatial instability is seen as non-zero spatial frequency (or number of bright blobs) around the ring. The spatial instability can be modeled, to the first order, using Gaussian beam propagation and interference considering a phase conjugate mirror^20^. This simple model predicts that the spatial frequency is $\pi kw^2/4d$ , where *d* is the distance between the $\chi ^{(3)}$ medium and the mirror, $w$ is the beam waist and *k* is the wavenumber. In addition, we also observed that the spatial frequency depends on the pump power that we associate to an effective angular momentum on the light induced by the polarization rotation inside the cell. The polarization mismatch of the counter-propagating pump fields (due to polarization self-rotation) induces an angular momentum on the standing wave^23,24^. This angular momentum is transferred to the scattered light resulting in an interference pattern along the ring. We observe a linear dependency of the spatial frequency on the pump power (see Fig. 3c), which is in agreement with the theory of polarization rotation where the ac-Stark shift changes linearly with the optical power.

The refractive index modulation, due to the standing-wave pump, coherently splits the scattering light propagating in opposite directions (i.e. the Bragg effect). Along with co- and counter-propagating four-wave mixing directions, this gives rise to the observation of two-mode and four-mode correlations in our system. Below, we first calculate the normalized intensity-difference noise of the AMWM signal for multiple spatial modes and compare it with the intensity-sum noise of the same two modes.

**Figure 4:**
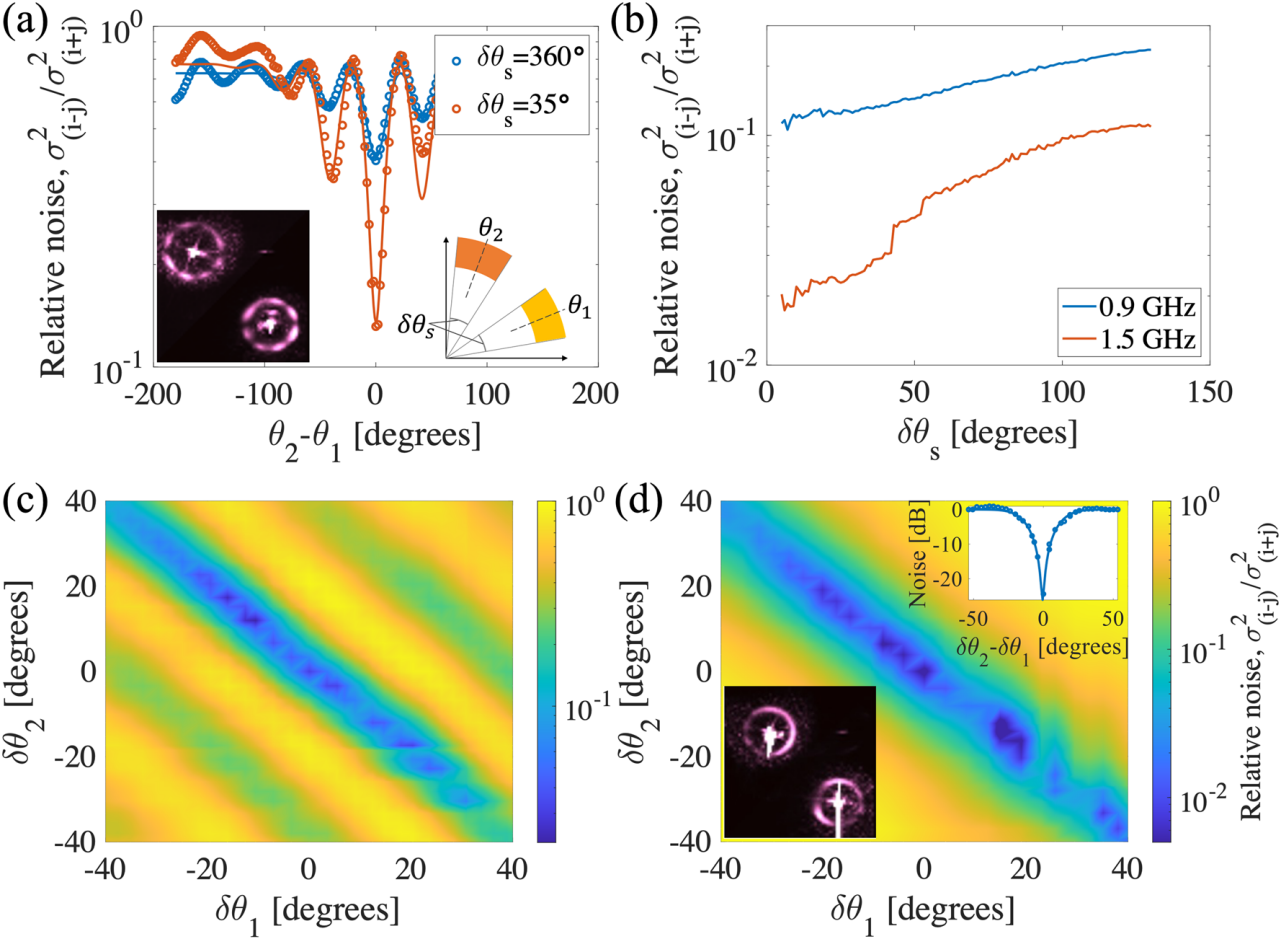
(**a**) Relative intensity noise, i.e. variance of the intensity difference relative to the variance of the intensity sum, as a function of the angular distance of $\theta _2-\theta _1$ for 0.9 GHz pump detunings. As shown in the inset on the right, angles $\theta _1$ and $\theta _2$ are angular centers of the analyzed segments for forward- and backward-propagating scattered light. $\delta \theta _s$ is the width of the analyzed segment as shown in the right inset in (**a**). The blue (red) data shows relative noise for $\delta \theta _s=35$ ($\delta \theta _s=360$) degrees. Data is fitted with a modulated Gaussian function. The inset on the left side shows images of the normalized counter-propagating ring signals. (**b**) Relative intensity noise as a function of analyzing angular segment width, $\delta \theta _s$. The blue (red) line shows the relative noise when the pump is 0.9 GHz (1.5 GHz) detuned from the lower excited state. (**c**) and (**d**) Two-dimensional plot of relative noise corresponding to detunings of 0.9 GHz and 1.5 GHz pump detunings, respectively. $\delta \theta _s$ in these plots are chosen to be as small as a single camera pixel. Inset on the right side of (**d**) is a diagonal cross-section of correlations fitted with a Gaussian profile.

We evaluated the noise in the intensity difference between different regions of the counter-propagating rings. We calculated the intensity-difference noise (or variance) normalized by intensity-sum noise, as a function of the relative angles between the rings. Figure 4 summarizes the results of the relative noise measurements between the AMWM signal, as a function of relative orientations. We defined the reference angles for each ring, $\theta _1$ and $\theta _2$, and digitally rotated one ring before subtracting the normalized intensities, to obtain the intensity-difference noise between the angular segments of the counter-propagating rings. This is achieved by first centering the two rings, and flipping one ring around its symmetry axis to align the correlated pixels of the two rings. By taking variance of the subtracted normalized intensity over time-sequenced images, we calculate the noise and normalize it to the variance of the intensity sum. To avoid influence of uneven intensity distribution over the rings, intensity of each pixel is normalized to temporal mean of that pixel. We can calculate the noise due to the total ring intensity, a segment of the ring, or a single pixel. We note that in this process we discard the saturated pixels.

The angular intensity correlation between counter-propagating rings is plotted in Fig. 4a for the relative noise in segments (or the entire area) of the rings. Each segment of the forward-propagating ring at an angle $\theta _1$ shows correlations with multiple segments of the counter-propagating rings centered at the $\theta _2$ angle. These correlated multiple segments match the periodic patterns of the rings.

Figure 4b shows the relative intensity noise as a function of the analyzed angular segment size. As also shown in Fig. 4a, smaller segments have more noise reduction than larger segments in both detunings. A noise reduction close to 20 dB is observed at the pump detuning of 1.5 GHz and using a small analyzing segment size.

Figure 4c,d show again the relative intensity noise for all segments (with different angular positions) of the forward- and backward-propagating rings at pump detunings of 0.9 GHz and 1.5 GHz, respectively. The segment size is chosen to be as small as a single pixel size. $\delta \theta _{1,2}$ is the angular displacement from the segment with the highest correlation. In Fig. 4d, the periodic pattern in the relative noise is not observed but there is high correlation between two symmetric modes.

To estimate the number of distinct spatial modes that show relative noise reduction with the counter-propagating modes, we calculate the intensity noise for a segment as small as a camera pixel. As shown in the inset of Fig. 4d, the intensity noise shows noise reduction over a small range of angles. We take the width of such intensity noise curve as the minimum angular width within which a single spatial mode can be defined. The total number of modes can then be derived by dividing the circumference of forward and backward rings ($2\times 2\pi$) by the full-width half-maximum of the intensity noise dip ($\delta \theta _{12}$). In this way, we find the mode capacity of the source to be more than 420.

**Figure 5:**
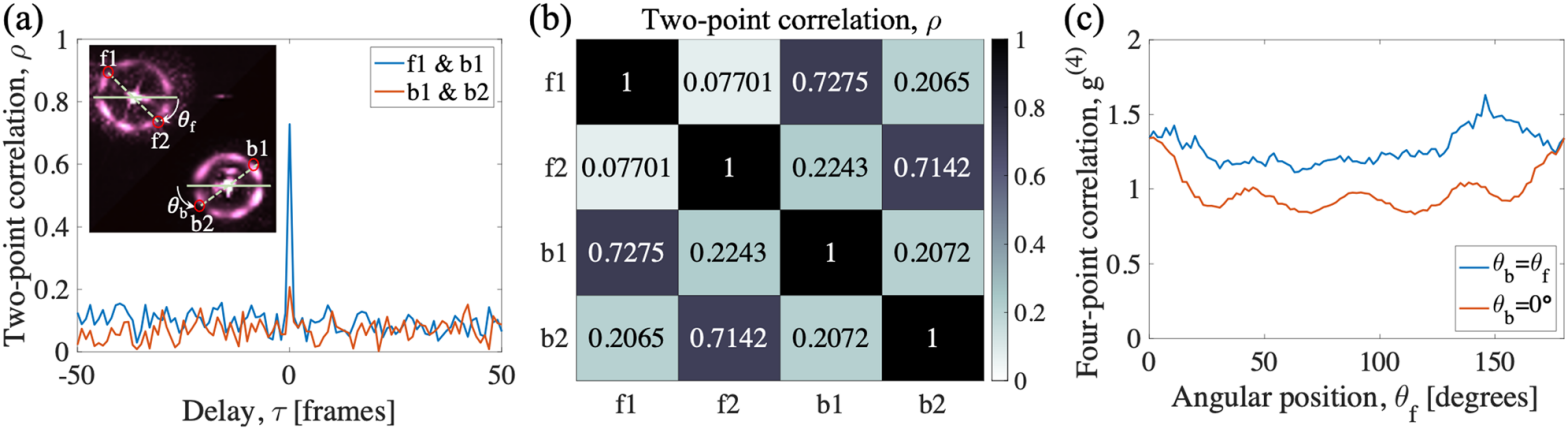
(**a**) Two-point intensity correlation is plotted for two sets of pixels as a function of delay in terms of the camera’s frame number. Inset shows EMCCD camera images for forward and backward AMWM signals. The bright center region is due to the pump leakage. The pixels indicated as *f*1, *f*2 and *b*1, *b*2 are two pixels of the forward- and backward-propagating AMWM signals and $\theta _{f/b}$ is the angular positions of these pixels from a fixed reference point. (**b**) Two-point correlations for zero time-delay (same frame) are shown for different combinations of four pixels shown in the inset of (**a**). (**c**) Four-point intensity correlation function at zero time delay, $g^{(4)}(0)$, is plotted as a function of angle $\theta _f$ as shown in the inset of (**a**). Consistent $g^{(4)}(0)>1$ is seen for many sets of four-pixel sets around the ring.

The correlation between two co-propagating or counter-propagating AMWM signals can be calculated using covariance normalized to variance values, $\rho =Cov(I_1,I_2)/\sqrt{Var(I_1)Var(I_2)}$. To evaluate the spatial correlations between different modes, the intensity of each pixel is normalized to the mean intensity of the frame. Figure 5a shows the correlation for two sets of co-propagating and counter-propagating modes. Highest correlation is observed at zero time (or frame) delay, because the long exposure time and slow frame rate of the EMCCD camera can not distinguish the short time delays between Stokes and anti-Stokes photons. The counter-propagating modes show high correlations, while co-propagating modes have small but positive correlations. Different absorption rates of Stokes and anti-Stokes photons can explain stronger correlation between counter-propagating modes^11^. When Stokes (anti-Stokes) photons are highly absorbed, only anti-Stokes (Stokes) photons will survive. This will lead to loss of correlation for photons propagating in the same direction while counter-propagating anti-Stokes (Stokes) photons maintain their correlations. Figure 5b shows the peak correlation value at zero delay (same frame) for different sets of forward and backward pixels (at fixed $\theta _{b/f}$) as shown in the inset of Fig. 5a. All pairs show larger-than-zero correlations, and counter-propagating modes show significantly higher correlations. We note that the backward image is flipped with respect to the vertical axis due to the geometry of the imaging setup. Also the intensity around the ring is asymmetric due to the asymmetric absorption of the Stokes and anti-Stokes signals^11^ and the alignment mismatch of the counter-propagating pump beams.

We also evaluate four-point correlations by calculating the 4th-order correlation function, $g^{(4)}$, for intensities of the segments at various angular positions (modes) around the ring. Figure 5c shows the result of $g^{(4)}(\tau =0)$ calculation for different angles. A consistent $g^{(4)}(\tau =0)>1$ can be seen for all angles when $f1 \& f2$ and $b1 \& b2$ pixel pairs are uniformly translated around the ring. The correlation drops when the relative angles $f1 \& f2$ and $b1 \& b2$ pixel pairs deviates from 0 or $\pi$. The fluctuation of $g^{(4)}(\tau =0)$ correlation corresponds to fluctuation of relative noise on Fig. 4. Considering the number of modes in the rings, 105 correlated quadruple modes are generated from the AMWM process. Classical correlation exists between all the bright spots in a same frame, but these quadruple modes show higher correlation. We expect phase matching condition of wave mixing process should allow quantum level correlation in these symmetric four mode. There is possibility that quantum correlation can exist between more than four modes by having another pair of counter-propagating pumps in different angle^5^.

**Figure 6:**
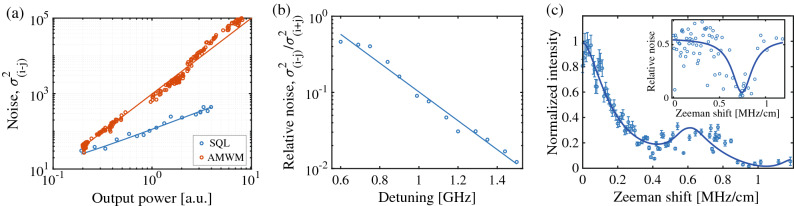
(**a**) Intensity-difference noise fitted with a quadratic model is shown as a function of the optical power for two modes of the four-wave mixing signal. The standard quantum limit (SQL) is measured using coherent laser beam pairs before the cell, and it is fitted with a linear line. (**b**) Relative noise as a function of pump detuning for $\delta \theta _s=35$ degrees with linear fitting. (**c**) Normalized intensity and relative noise (inset) of the scattered ring signal as a function of the magnetic field gradient. The solid line in the main plot is the theory (see the main text) using typical experimental values (e.g. $\Delta =200\gamma$, $\Omega =4\gamma$, $\gamma _0=0.04\gamma$), and the solid line in the inset is a Lorentzian curve as a guide to the eye.

The noise reduction reported above is relative to the intensity noise of the Stokes and anti-Stokes light. To compare the intensity-difference noise with the standard quantum limit (SQL), we split the laser before the cell and measure the intensity-difference noise of the coherent laser light for varying power. We also measured the intensity-difference noise for the AMWM signal after the cell. The results are shown in Fig. 6a. AMWM noise was fitted with a quadratic model and the SQL was fitted with a linear model. We note that the intensity-sum noise also shows quadratic dependency with the pump power. This suggests that the intensity-difference noise is limited by the integration time of the camera that is much larger than the inverse scattering rate. The AMWM signal is relatively bright and each camera frame contains thousands of photons whose noise is averaged before subtraction from the corresponding frame of the correlated pixel. At low powers the AMWM noise approaches the SQL. A faster camera should enable clear observation of intensity squeezing between multiple modes. We note that such intensity squeezing is different from the seeded four-wave mixing^25–28^ where an incident probe is amplified and a correlated conjugate is created. Also, the amplified nature of the multi-wave mixing process enables observation of bright Stokes and anti-Stokes signals with lower pump powers compared to the spontaneous four-wave mixing process^29,30^. We also observed that the relative noise exponentially decreases with the pump frequency within a range of pump detunings (see Fig. 6b). At larger detunings, incoherent Raman scattering and re-absorption is reduced leading to less noise. We note that just outside the frequency range plotted in Fig. 6b, the scattering ring could not be observed but it reappeared again when the pump frequency reached the next detuning window supporting AMWM.

Another factor limiting the degree of correlation is single-photon or two-photon re-absorption of Stokes or anti-Stokes photons. This effect can be reduced applying an atomic frequency gradient^31^. To model the effect of the external broadening on re-absorption, we write Maxwell-Bloch equations and solve for Stokes and anti-Stokes field in presence of a two-photon detuning gradient^31^, $\delta (z)=\eta z$, along the propagation direction, *z*. Considering a pump light detuning much larger than the excited state linewidth, i.e. $\Delta \gg \gamma$, we can model Stokes ($\mathcal {E}_S$) and anti-Stokes ($\mathcal {E}_{aS}$) fields propagation using the following Maxwell equation:

1 $$\begin{aligned} \frac{\partial }{\partial z}\left( \begin{array}{c} \mathcal {E}_S(z) \\ \mathcal {E}^*_{aS}(z) \end{array}\right) =i a_0\left( \begin{array}{cc}a_{11} &{} a_{12} \\ a_{21} &{} a_{22} \end{array}\right) \left( \begin{array}{c} \mathcal {E}_S(z) \\ \mathcal {E}^*_{aS}(z) \end{array}\right) \end{aligned}$$

where $a_0$ is proportional to the optical density and $a_{11}$, $a_{12}$, $a_{21}$ and $a_{22}$ are effective coupling strengths written in terms of pump Rabi frequency, $\Omega$, detuning $\Delta$, excited-state decay rate, $\gamma$, and decoherence rate, $\gamma _0$^31^.

To introduce the atomic frequency gradient, we apply a linearly varying Zeeman broadening using a solenoid. Figure 6c shows the measured intensity and intensity noise of two modes of the AMWM signal as a function of the magnetic field gradient. The intensity drops as the induced field gradient reduces the effective optical density. On the other hand, the corresponding reduction in the re-absorption due to the B-field gradient causes enhanced scattering and reduced intensity noise at a gradient of about 0.6 MHz/cm, which corresponds to about one $\gamma$ of induced broadening. This agrees with the model for the anti-Stokes intensity, $|\mathcal {E}^*_{aS}|^2$, plotted using the equation above. We also coupled two counter-propagating correlated modes to two single-mode fibers and used single-photon detectors (SPD) to measure $g^{(2)}$ function. However, we observed that the strong AMWM signal and the background pump scattering saturates the SPD, even at the lowest power point in Fig. 6a. At lower powers, the amplification and instability could not be observed.

To extract the maximum correlations between different spatial modes, the exposure time of the camera should be shorter than the coherence time or the inverse of the Raman scattering bandwidth, whichever is smaller. The photons arriving at different times within a single integration time (a single camera frame) are not expected to have correlations and they reduce the strength of noise reduction in the measurement. The lack of effective noise subtraction is evidenced by quadratic dependency of both intensity-sum and intensity-difference noise as a function of mean intensity. Therefore, the correlation measurement described above is an underestimation of the maximum correlations that exists in the system. The relatively large exposure time of the camera cannot be compensated for by using lower pump powers to reduce the scattering rate. Below the threshold pump power, the amplification could not be observed, and also at low scattering rates, the electronic noise of the camera overcomes the shot noise. Also, a camera with higher spatial resolution images will allow us to more accurately determine the maximum correlation and mode capacity of the source. By using two similar cameras (one for each ring) or one camera with higher temporal and spatial resolutions, larger correlations can be extracted from the images.

The study reported here sheds light on some complex mode dynamics, transition to instability, and correlations in driven dispersive nonlinear resonators. The multimode and spatially-distinct nature of the correlations in the AMWM studied here can also find applications in study of phase transition^17^, multimode optical switching^18^, quantum imaging^32,33^, and quantum communications^7,34^ using room-temperature sources. For example, by reflecting one beam from an object and detecting both correlated beams on a CCD camera, the intensity correlations can be used to measure change in the reflectivity beyond the state-of-the-art classical devices^35^.

## Data Availability

The data used and presented in this study are available upon reasonable request, by the corresponding author.
